# DNA methylation and expression of proopiomelanocortin (*POMC*) gene in the hypothalamus of three‐week‐old chickens show sex‐specific differences

**DOI:** 10.1002/2211-5463.12427

**Published:** 2018-04-27

**Authors:** Rebecca C. Rancourt, Karen Schellong, Barbara Tzschentke, Wolfgang Henrich, Andreas Plagemann

**Affiliations:** ^1^ Division of ‘Experimental Obstetrics’ Clinic of Obstetrics Charité – Universitätsmedizin Berlin corporate member of Freie Universität Berlin Humboldt‐Universität zu Berlin, and Berlin Institute of Health Germany; ^2^ Institute of Biology Humboldt‐University of Berlin Germany; ^3^ Clinic of Obstetrics Charité – Universitätsmedizin Berlin Germany

**Keywords:** chicken, DNA methylation, epigenetics, hypothalamus, proopiomelanocortin, sex specific

## Abstract

Increased availability and improved sequence annotation of the chicken (*Gallus gallus* f.* domestica*) genome have sparked interest in the bird as a model system to investigate translational embryonic development and health/disease outcomes. However, the epigenetics of this bird genome remain unclear. The aim of this study was to determine the levels of gene expression and DNA methylation at the proopiomelanocortin (*POMC*) gene in the hypothalamus of 3‐week‐old chickens. *POMC* is a key player in the control of the stress response, food intake, and metabolism. DNA methylation of the promoter, CpG island, and gene body regions of *POMC* were measured. Our data illustrate the pattern, variability, and functionality of DNA methylation for *POMC* expression in the chicken. Our findings show correlation of methylation pattern and gene expression along with sex‐specific differences in *POMC*. Overall, these novel data highlight the promising potential of the chicken as a model and also the need for breeders and researchers to consider sex ratios in their studies.

**Abbreviations**
NI: nucleus infundibuli hypothalami
*POMC*: proopiomelanocortin
qPCR: quantitative real‐time PCR

The chicken (*Gallus gallus* f.* domestica*) provides a unique opportunity to study various health conditions and environments when addressing developmental origins of health and diseases/‘perinatal programming’ 1. In particular, due to the independent development from the mother, the chicken embryo provides a valuable model to distinctively establish causal factors and mechanisms. Researchers have been effectively using the chicken for various physiological investigations into embryonic developmental time points 2, 3, for example, retinal 4, neuronal and endocrine system 5, 6, 7, as well as a model for studying other health outcomes such as the metabolic syndrome 8, 9, 10, 11. There is growing interest in the field of epigenetics to fully characterize and understand the mechanistic manner through which environmental factors during embryonic/fetal development or other important time points, for example*,* puberty, can influence the expression of genes as well as affecting downstream health outcomes 12, 13. However, this research into the epigenome has predominately been on mammalian genomes such as human and rodents 14, 15. Increased availability and improved sequence annotation of this bird genome have allowed the chicken to rise as a model system to investigate these topics 16, 17. Recent genome‐ and transcriptome‐wide analyses pertaining to epigenetic mechanisms such as DNA methylation have further highlighted the similarities and important differences across species 4, 9, 18, 19, 20, 21, 22, 23, 24. There are differences in epigenetic mechanisms in chicken *versus* mammals such as the allele‐specific phenomena of genomic imprinting, which has not been observed in chicken 18, 25, 26, 27. Another difference is, for instance, the chicken sex‐determination chromosome when compared to mammals. In mammals, females have two homogeneous (XX) sex chromosomes and males have heterogeneous (XY) sex chromosomes. In contrast, for birds, the male has two homogeneous (ZZ) sex chromosomes and the female has heterogeneous (ZW). Additionally, the chicken sex chromosomes have different gene clusters and arrangements/positions due to evolutional divergence 28, 29.

Despite the growing amount of research/knowledge into the chicken epigenome, the underlining molecular mechanisms driving epigenetic regulation in bird are still not clearly defined or characterized. We aimed to provide insight into the central nervous/hypothalamic expression of proopiomelanocortin (*POMC*), a neurohormone with important physiological roles, for example, for food intake and body weight control (reviewed in ref. 30), and examine the methylation profile at the CpG sites across the promoter region and the CpG island in the gene body of *POMC* in 3‐week‐old chickens. Additionally, we specifically chose to use brain samples in early adolescence, prior to the occurrence of sexual dimorphism in chicken 31, in order to identify sex‐specific differences and influences as we narrowed in on the control center for temperature and food intake//body weight regulation with a gene‐targeted approach.

## Materials and methods

### Ethics statement

All animal procedures were performed in accordance with the European Communities Council Directive (86/609/EEC) and were approved by the local animal welfare committee (G 0275/09; Lageso Berlin, Germany).

### Animal model and study design

Experiments were carried out on microdissected brain samples of 3‐week‐old juvenile chickens (*Gallus gallus* f.* domestica*), hatched from eggs which were obtained for research approaches. The eggs were purchased from a commercial breeder (Lohmann Tierzucht GmbH, Cuxhaven, Germany) and incubated 21 days in our laboratory under standard conditions (37.5 °C, relative air humidity 70–90% during hatching period, automatically turning up to day 18 of incubation) 8, 10. Chickens were housed under standardized environmental and alimentary conditions (ambient temperature of 25 °C with relative air humidity of 30%) during 3 weeks of life. An infrared lamp was an additional source of heat (35 °C) for the chicks until day 14 post‐hatching. Food (complete feed, ssniff Spezialdiäten, Soest, Germany) and water were provided *ad libitum* to all animals.

### Sample preparation

For molecular biology analyses, the nucleus infundibuli hypothalami (NI) was microdissected from deep‐frozen brain slices 8, 15. Genomic DNA and total RNA were simultaneously isolated from the NI brain probes using the ZR‐Duet™ DNA/RNA MiniPrep Kit (Zymo Research, Irvine, CA, USA) according to the manufacturer's instructions. cDNA was synthesized from total RNA according to the manufacturer's protocol of the iScript™ cDNA Synthesis Kit (Bio‐Rad, Hercules, CA, USA), and reverse transcriptase minus (RT−) negative controls were included. Genomic DNA was bisulfite treated using the EZ DNA Methylation‐Gold Kit (Zymo Research) following manufacturer's protocol.

### Gene expression analysis

Quantitative real‐time PCR (qPCR) was used to measure the relative mRNA expression for the gene, *POMC*, similar to as described in Rancourt *et al*. 8. Commercially available TaqMan^®^ probe‐based gene expression assays were used (Life Technologies, Carlsbad, CA, USA) and were run on an Applied Biosystems 7500 instrument according to the manufacturer's protocol (Applied Biosystems, Waltham, MA, USA). Expression levels were normalized to the housekeeping gene *BETA ACTIN*. When possible exon‐spanning primer sets were selected, qPCR was performed as duplex qPCR with housekeeping gene. Assays were carried out in triplicate, and relative gene expression was calculated using the $2^{-\Delta C_{T}}$ method corrected for the amplification efficiency calculated from standard curves for all primer sets 8, 15, 32. TaqMan^®^ gene expression assays: *POMC*: Gg03352057_m1 and *BETA ACTIN*: Gg03815934_s1, VIC‐labeled, primer limited.

### DNA methylation assays

Target regions which included promoter regions, CpG islands, and gene body for pyrosequencing analyses were selected with UCSC genome browser (build: Chicken Nov. 2011, ICGSC Gallus_gallus‐4.0/galGal4) as described in Rancourt *et al*. 8. UCSC annotated CpG islands were further confirmed with CpGPlot (http://www.ebi.ac.uk/Tools/seqstats/emboss_cpgplot/). Methylation assays were designed using the pyromark Assay Design Software 2.0 (Qiagen, Valencia, CA, USA, http://www.qiagen.com). Bisulfite‐converted DNA was mixed with 0.2 μm of each primer and amplified using either HotStarTaq plus Master Mix (Qiagen) or ZymoTaq (Zymo Research) following standard procedures. The Pyromark Q24 pyrosequencer (Qiagen) was used for pyrosequencing on PCR amplicons. Percent methylation was analyzed across individual CpG sites located within the regions of interest at chromosome 3 *POMC* locus covering 17 CpG sites. All assays included a bisulfite conversion check to verify full conversion of the DNA, and assays were validated with a methylation scale (0–100%). Primer sequences and pyrosequencing assay information are provided in Table 1.

**Table 1:** Pyrosequencing assay information

Target region	Primers	5′–3′ sequence	Chromosomal location^a^	*T*_m_ (°C)
POMC promoter	Forward biotinylated	GTAGGGGTTGTAGTTTGTAGGTA	chr3: 105 014 771–105 015 196	59.1
	Reverse	ACCAAATCCTAACACTTACTATTCTC		59.7
	Sequencing S1	CCCAAATCCTTTATCACCTA		
	Sequence to analyse S1	CRTAAACACCCRACTTTACAAATAACAACTACTACCRT		
	Unconverted sequence S1	ACGGCAGCAGCTGTCACCTGCAAAGCCGGGTGTTCACG		
	Sequencing S2	AACAACCCCAACACC		
	Sequence to analyse S2	ACRAACAAACTATAACACAACRCRCCCCRCATCCTACTAACRAAAAATAAACACCCAAACTATAAAAAAACTATAAAAAAA		
	Unconverted sequence S2	TTTTCCCATAGCTCCTCCACAGTTTGGGTGCCCACCCCTCGCCAGTAGGATGCGGGGCGCGCTGTGCCACAGCCTGCCCGT		
POMC CpG island – Gene body	Forward biotinylated	AAGATGGAGAAGGGTTGGAA	chr3: 105 016 280–105 016 576	58.2
	Reverse	AAATCTAACTATACTCCAAACTCA		56.5
	Sequencing S1	AACTCCATAAAATAACTCTCAA		
	Sequence to analyse S1	CCRACTCCTCRTCCACCCCRTTAAAATACACCTTAATAAATCTCC		
	Unconverted sequence S1	CGGGGTGGACGAGGAGTCG		
	Sequencing S2	CTCCAAACTCATAAAAC		
	Sequence to analyse S2	CRCCRTAACRCTTATCCTTCAACRACRCRTAC		
	Unconverted sequence S2	GCACGCGCCGCTGAAGGACAAGCGCTACGGCG		

a Chromosomal location is based on the UCSC Chicken Nov. 2011 (ICGSC Gallus_gallus‐4.0/galGal4) Build.

### Statistical analyses

Normal/healthy randomly selected animals were used for analysis, and the highest available number of sample measurements is presented here. Real‐time data are given as arbitrary units. For statistical analyses of the investigated real‐time expression and pyrosequencing methylation data concerning differences between groups, Student′s *t*‐test for independent samples (if normally distributed) or Mann–Whitney *U*‐test (if not normally distributed) was used. Significance level was set at *P < *0.05. For analyses of relations between two variables, Spearman's rank correlation test was performed overall and by groups. All statistical tests were carried out with graphpad prism (version 4.03, San Diego, CA, USA).

## Results

### DNA methylation levels across the genomic landscape of the chicken *POMC* gene

**Figure 1:**
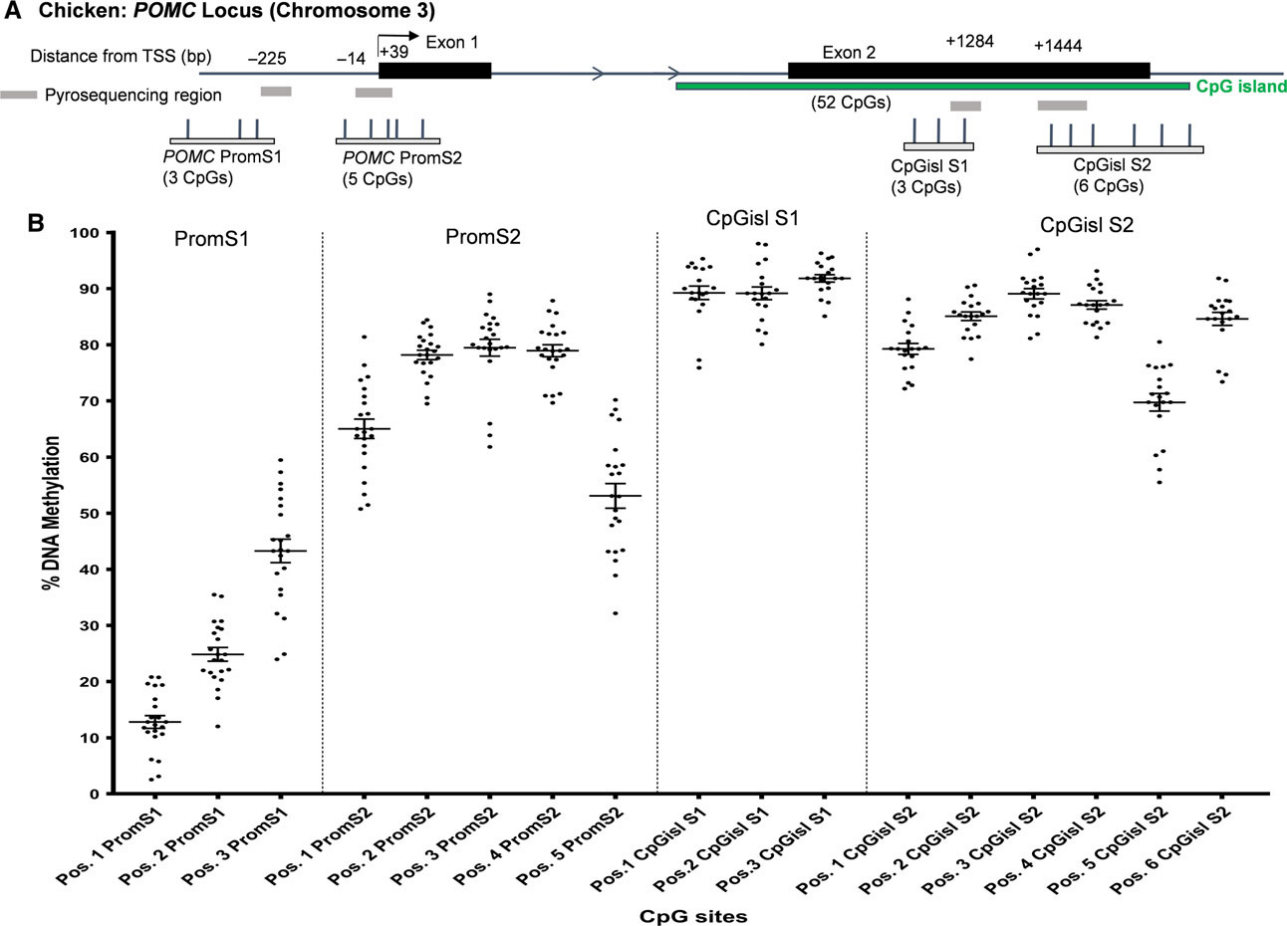
Chicken POMC locus and DNA methylation analyses. (A) Schematic representation of the sequencing map for the POMC gene region including CpG islands, promoter, and gene body chromosomal locations of pyrosequencing assays. (B) Corresponding DNA methylation levels at individual CpG sites across the target regions in 3‐week‐old juvenile chickens (Gallus gallus f. domestica). n = 21.

An interesting pattern was observed in the chicken *POMC* promoter region, in which the levels across the eight sites ranged from ~ 2% to 90% (average 54% methylation across the eight sites at the promoter area), and the intrachicken values at each CpG site had a variation of 20–40% (Fig. 1). Hypomethylation/low levels were measured at the first two CpG sites (Pos1. PromS1, average 13% and Pos2. PromS1, average 25%), while moderate methylation levels (40–60%) were observed at sites Pos3. PromS1; Pos1. PromS2; and Pos5. PromS2, and hypermethylation/high levels (> 75%) occurred at sites Pos.2‐4 PromS2. Across nine sites in the CpG island within the gene body, the levels ranged from 55% to 98% with an overall average of 85% and exhibited an overall more hypermethylation profile (Fig. 1).

### Sex‐specific observations and correlation analyses

**Figure 2:**
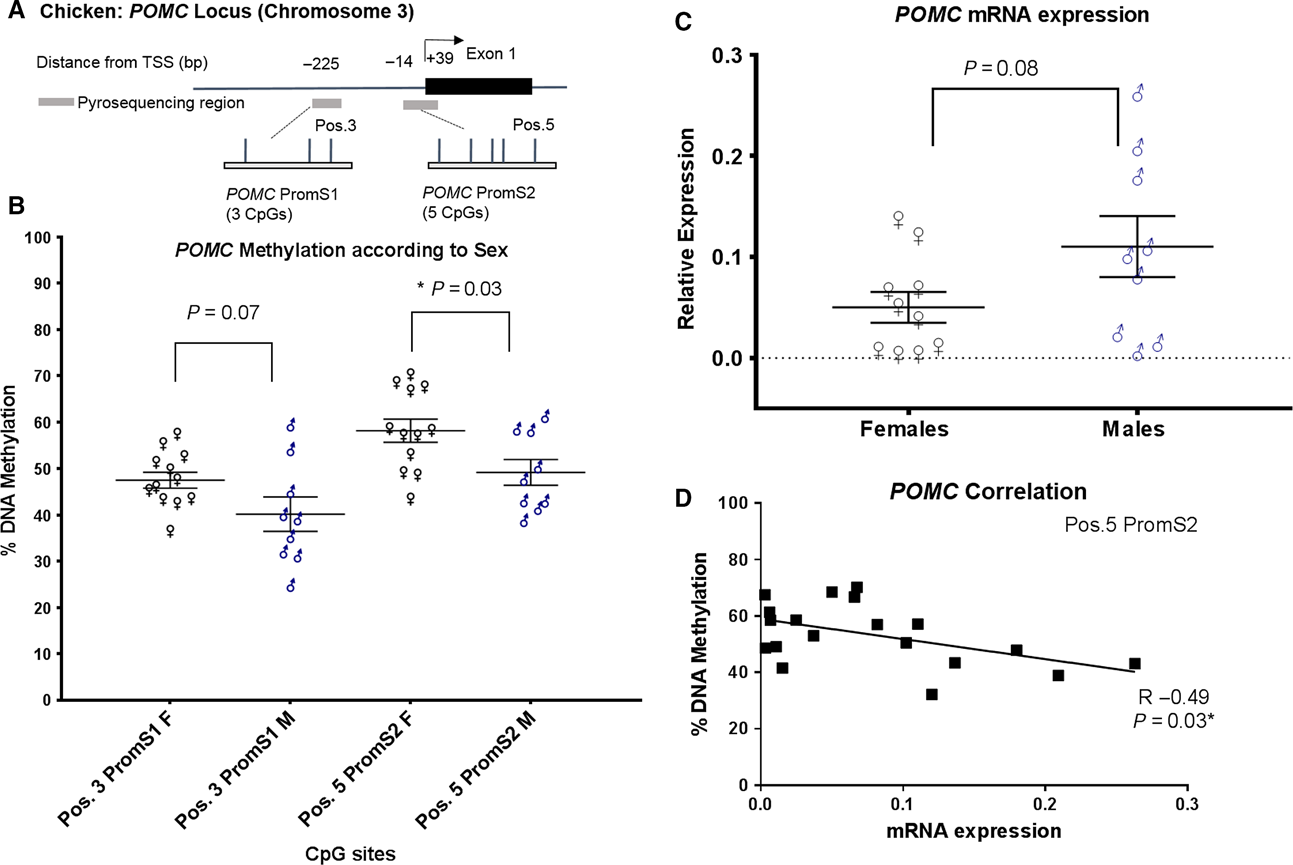
Hypothalamic POMC DNA methylation and gene expression according to sex and correlation analysis in 3‐week‐old chickens. (A) Schematic representation of the POMC promoter region pyrosequencing assays. (B) Sex‐specific differences for DNA methylation levels at POMC promoter region CpG sites Pos.3 PromS1 and Pos.5 PromS2. F, females; M, males. (C) Hypothalamic POMC mRNA expression according to sex. (D) Statistically significant relation between DNA methylation level at promoter CpG site, Pos.5 PromS2, and mRNA expression of POMC. n = 21.

Sex‐specific differences in methylation levels were observed at two CpG sites in the *POMC* promoter region with females having higher methylation than males (Pos.3 PromS1 47% in females *versus* 40% in males *P* = 0.07 and at Pos.5 PromS2 58% in females *versus* 49% in males *P* = 0.03 Fig. 2B, Table 2). The sex‐specific difference in *POMC* DNA methylation compliments the trend in *POMC* gene expression with females having lower mRNA expressions than males (*P* = 0.08, Fig. 2C, Table 2). Accordingly, an inverse correlation of mRNA expression *versus* DNA methylation was seen in the promoter target region at CpG site Pos.5 PromS2 (*R* *=* −0.49, *P* = 0.03, Fig. 2D). No differences were observed in total body weight according to sex at the time points of day 1, 1, 2, or 3 weeks of age (Table 2).

**Table 2:** Chicken characteristics, hypothalamic *POMC* gene expression, and DNA methylation according to sex

Variables	Females	Males	*P*‐value
Body weight development (g)			
Day 1	39.95 ± 0.96 (12)	39.94 ± 0.92 (9)	0.85
1 Week	72.1 ± 2.69 (12)	69.5 ± 1.81 (9)	0.46
2 Weeks	146.4 ± 5.78 (12)	145.9 ± 4.48 (9)	0.95
3 Weeks	242.4 ± 9.61 (12)	249.9 ± 7.72 (9)	0.57
*POMC* mRNA expression (arbitrary units)	0.05 ± 0.01 (10)	0.11 ± 0.03 (9)	0.08
*POMC* target regions methylation (%)			
Promoter region			
Pos.1 PromS1	13.07 ± 1.73 (12)	14.35 ± 2.55 (9)	0.67
Pos.2 PromS1	27.66 ± 2.44 (12)	23.18 ± 1.38 (9)	0.16
Pos.3 PromS1	47.55 ± 1.72 (12)	40.22 ± 3.72 (9)	0.07
Pos.1 PromS2	65.37 ± 2.36 (12)	64.61 ± 2.97 (9)	0.84
Pos.2 PromS2	76.55 ± 1.56 (12)	79.05 ± 1.20 (9)	0.25
Pos.3 PromS2	77.26 ± 2.53 (12)	82.8 ± 0.79 (9)	0.08
Pos.4 PromS2	78.52 ± 1.42 (12)	79.49 ± 1.86 (9)	0.67
Pos.5 PromS2	58.13 ± 2.51 (12)	49.24 ± 2.78 (9)	**0.03**
CpG island gene body region			
Pos.1 CpGislS1	86.66 ± 2.28 (9)	90.77 ± 1.14 (9)	0.13
Pos.2 CpGislS1	88.4 ± 1.76 (9)	89.22 ± 1.84 (9)	0.75
Pos.3 CpGislS1	90.42 ± 1.18 (9)	92.39 ± 0.96 (9)	0.21
Pos.1 CpGislS2	79.03 ± 1.19 (9)	79.04 ± 1.82 (9)	0.99
Pos.2 CpGislS2	85.3 ± 1.42 (9)	84.42 ± 0.95 (9)	0.62
Pos.3 CpGislS2	89.28 ± 1.76 (9)	88.38 ± 1.11 (9)	0.67
Pos.4 CpGislS2	85.78 ± 1.36 (9)	87.59 ± 1.04 (9)	0.30
Pos.5 CpGislS2	68.76 ± 2.67 (9)	70.08 ± 2.14 (9)	0.61
Pos.6 CpGislS2	82.97 ± 1.92 (9)	86.88 ± 0.80 (9)	0.08

Values are expressed as means ± SEM. Number of animals in parentheses. *P*‐values calculated using Student's *t*‐test or Mann–Whitney *U*‐test when appropriate. Significance level was set at P < 0.05 (as shown in bold).

## Discussion

The overall aim was to provide the first characterization of the chicken (*Gallus gallus* f.* domestica*) epigenetic profile and transcription/expression of *POMC* in order to contribute to the growing research of this practical and reliable model system. The chicken offers many versatile possibilities for investigating embryonic development especially considering it is a ‘closed’ developmental system and gestation takes only 21 days. We measured methylation levels across 17 CpG sites in the *POMC* gene, 8 sites encompassing the promoter region and the remaining 9 sites in a CpG island within the gene body. The promoter CpG sites showed an interesting pattern with a variation in DNA methylation levels. Other investigations have similarly observed ranging methylation levels in other target genes; for example, we previously observed this occurring at the gene promoter regions for glucose transporter 1 and insulin receptor precursor in the chicken 8, 21. The higher methylation levels observed at the CpG island are what is typically reported within gene body regions. Notably, the chicken promoter region is less conserved across species as compared to the CpG island gene body region, which exhibits more conservation across species (taken from the conservation track at UCSC, 33). It has been reported that the chicken NI region is very similar to the Nucleus arcuatus hypothalami (ARC) in mammals (*e.g.,* human and rodents) 34, 35. Franke *et al*. 36 measured the ratio of *Pomc* expressing cells in the ARC of 3‐week‐old control rats to be around 20%. While it is difficult to extrapolate the exact ratio in the present study, a uniform area was dissected in the *POMC*‐relevant NI region. Cellular mixtures could possibly explain the interesting mid‐ranged methylation levels across some CpG sites examined (*e.g.,* ranges 23–70%).

To the best of our knowledge, we are the first to report DNA methylation profiles at the *POMC* promoter and CpG island/gene body regions in the chicken hypothalamus. We observed clear sex‐specific differences in DNA methylation pattern at the *POMC* promoter region and gene expression. This was accompanied by distinct correlation between gene expression and promoter methylation. Accordingly, our data suggest that DNA methylation levels at specific CpG sites in the promoter region are influencing the hypothalamic *POMC* mRNA expression in a sex‐specific manner. However, the exact underpinnings of how this regulation is influencing outcomes or what possible phenotypic consequences may result are still unknown. Despite that at the time of molecular analysis (i.e., DNA methylation and gene expression), there were no weight differences according to sex, perhaps weight differences or other phenotypic differences related to observed epigenetic pattern could appear at a later time point of life. Typically differences in sex‐specific expression have been known to occur with gene dosage *via* gene copies on sex‐linked chromosomes (e.g., mammals: X, Y; and in birds: Z, W) although this cannot be explained here in this case as *POMC* is not on the Z or W chromosome. Other studies involving chicken, for example, those performed by Warnefors *et al*. 37 illustrated sex‐specific differences in microRNA expression describing microRNAs as the gene‐specific dosage compensation mechanism. Additionally, Nätt *et al*. 31 reported sex‐specific differences in genomewide analyses with promoter DNA methylation appearing to affect sex‐specific expression in a site/gene‐specific manner.

Differences in the establishment of the *POMC*‐related hypothalamic processes could contribute to setting up variations in regulated phenotypic, especially vegetative functions throughout later life (e.g., growth trajectories, total and/or abdominal fat acquisition, stress response), and this may be programmed according to sex. Our results showing hypothalamic DNA methylation and gene expression differences in a key physiological player, *POMC*, suggest the chicken as a positive/promising model having great potential for interrogating the underpinnings for, for example, obesity in humans. Taken together, for the first time the provided data illustrate the pattern, variability, and functionality of DNA methylation for *POMC* expression in the chicken. The findings of sex‐specific differences point to the importance for researchers and breeders to consider the sex ratios/differences in respective studies.

## Author contributions

RCR, KS, BT, and AP conceived and supervised the study; RCR, KS, BT, and AP designed experiments; RCR and KS performed experiments; BT, AP, and WH provided equipment, samples, and reagents; RCR, KS, and AP analyzed data; RCR and AP wrote the manuscript; RCR, KS, BT, WH, and AP reviewed and revised manuscript.
